# Mortality among workers exposed to asbestos at the shipyard of Genoa, Italy: a 55 years follow-up

**DOI:** 10.1186/s12940-018-0439-1

**Published:** 2018-12-29

**Authors:** Domenico Franco Merlo, Marco Bruzzone, Paolo Bruzzi, Elsa Garrone, Riccardo Puntoni, Lucia Maiorana, Marcello Ceppi

**Affiliations:** 1 Research and Statistics Infrastructure, Azienda Unità Sanitaria Locale – IRCCS di Reggio Emilia, Institute for Advanced Technologies and Health Care Models in Oncology, Reggio Emilia, Italy; 2 Clinical Epidemiology, Ospedale Policlinico San Martino-IRCCS, Istituto di Ricovero e Cura a Carattere Scientifico per l’Oncologia, Genoa, Italy; 3 Environmental Epidemiology, Ospedale Policlinico San Martino-IRCCS, Istituto di Ricovero e Cura a Carattere Scientifico per l’Oncologia, Genoa, Italy

**Keywords:** Asbestos, Mortality, Shipyard, Mesotheliomas, Cancer

## Abstract

**Background:**

Exposure to asbestos remains a global issue due to its massive use in the twentieth century and its long environmental persistence. Exposure to asbestos still occurs during dismantling of ships and vessels, buildings renovation, mining operations, and is reported in developing countries. Current estimate report exposure of hundreds of million people in occupational settings in countries where its use remains unregulated.

**Methods:**

We conducted a historical prospective cohort mortality study aimed at estimating mortality from specific causes, the temporal changes of pleural and lung cancer mortality, and the attributable fraction (AF) of lung cancer deaths following asbestos exposure. The study included 3984 shipyard workers employed at the shipyard of Genoa, Italy, between 1960 and 1981 and followed up to December 2014. Standardized Mortality Ratios (SMR) and their 95% confidence intervals (95%CI) were computed.

**Results:**

Overall deaths recorded were 3331 (83.6%). Excess mortality was observed for all cancers (SMR = 127, 95%CI:120-134), pleural mesothelioma (575, 469–697), cancers of the larynx (183, 134-244) and of the lung (154, 139-170), and for respiratory tract diseases (127, 114-141), including asbestosis (2277, 1525-3270). Ninety out of 399 deaths (22.6%) from lung cancer were attributed to asbestos exposure. The estimated lung cancer AF was 49.3% in workers with the highest SMR for pleural cancer. Median latency times for pleural and lung cancer were 42.8 years (minimum latency: 9.3 years) and 38.7 years (minimum latency: 6 years). The peak of mesothelioma incidence, expected in Italy in the period 2015–2024, was confirmed.

**Conclusions:**

The long follow-up period of our study allowed the detection of a substantial disease burden following asbestos exposure. These findings support the urgent need for the prevention of asbestos related diseases through the implementation of asbestos ban worldwide, including those countries where asbestos is still mined, manufactured and used.

## Background

The association between occupational exposure to asbestos fibers and health impairment have long been reckoned, with the first historical source found in a writing by Pliny the Younger dating back to 50 A.D [1]. Since then, it was only in the twentieth century that this association returned under scrutiny when in 1924 the British Medical Journal published the case of a deceased 33-year old woman who had worked in an asbestos textile factory for 17 years and whose lungs presented a macroscopic and microscopic fibrotic appearance [2]. A few years later asbestos was indicated as the cause of extensive lung fibrosis that was named asbestosis, a fatal chronic lung disease [3]. During the 1960s and early 1970s asbestos exposure was unequivocally linked by a series of epidemiological and experimental studies to the risk of malignant pleural mesothelioma, a rare and aggressive tumor arising from the mesothelial cells lining the pleural cavity and that can develop in the peritoneum, the pericardium and the tunica vaginalis. Indeed, mesothelioma was shown to be almost exclusively linked by exposure to asbestos, with an aetiological fraction ≥80% [4]. Increased risks of mesothelioma have been reported among asbestos miners and workers, insulation and shipyard workers, in people living near asbestos factories, mines and shipyards, and in relatives of asbestos workers [5–18]. A recent pooled mortality analysis of 43 Italian cohorts of workers exposed to asbestos followed up between 1970 and 2010 confirmed increased mortality for pleura and peritoneal mesothelioma and lung cancer [19]. In particular, mortality rates from pleural cancer showed an increasing trend during the first 30 years since first exposure to asbestos, which then reaches a plateau thereafter.. A study of four Finnish asbestos-exposed cohorts followed up between 1967 and 2012 revealed 3 to 100-fold increased incidence for mesothelioma and 2 to 10-fold for lung cancer [20]. The Swedish component of the Nordic Occupational Cancer study (NOCCA) reported increased risk of mesothelioma with a clear dose-response relationship [21]. Dose-related trends of asbestos exposure and incidence of all cancers, esophageal and respiratory tract cancers were reported among Taiwanese shipbreaking workers [22].

The projected mortality of men dying from mesothelioma for the period 1995–2019 in seven Western European countries, predicted a twofold increase of deaths over the next two decades (from 5000 in 1998 to about 9000 around 2018), followed by decline [23].

Inhaled asbestos is also a known risk factor for lung cancer acting synergistically with smoking [24–26]. Morover, excess mortality for laryngeal cancer has been reported in workers with any exposure to asbestos (+ 40%) and in highly exposed subjects (+ 160%) [27] and according to the International Agency for Research on Cancer (IARC) there is sufficient evidence of asbestos causation of laryngeal cancer [28]. A review of cancer risk among shipyard workers concluded that asbestos was the main source of excess cancer risks for malignant mesothelioma, cancer of the lung and larynx [29].

A mortality study was conducted in workers employed at the shipyard of Genoa, Italy, employed or retired between 1960 and 1981and followed up to the end of 1995, and reported increased mortality for pleural, lung, larynx and bladder cancers, as well as for respiratory diseases [30]. With specific reference to Italian shipyard workers who were assigned mainly to ship repair, refitting and construction, exposure was especially elevated as asbestos was used to insulate boilers, steam and hot water pipes, and as an insulator for the air conditioning systems. Asbestos was present in construction sheets and also sprayed directly on surfaces. Shipyard workers were exposed to a variety of toxic agents other than asbestos, including solvents, welding fumes, polycyclic aromatic hydrocarbons, and metal-working fluids [31], In Italy, asbestos has been in use since 1920 –especially in the construction and shipyard sectors– up until the seventies first with the introduction of regulations and protective measures in the shipyard field [31], and later in 1992 with the total ban on the use of asbestos nationwide [32].

The present study further extended the cohort follow-up of by 19 years, yelding an overall 55-year observation period. The prolonged exposure to asbestos fibers experienced at the shipyard and the long follow-up period represented a unique opportunity to investigate asbestos health effects, changes in mortality rates for pleural mesotheliomas and lung cancers across the follow-up window, and quantify the excess mortality for lung cancer attributable to asbestos exposure.

## Methods

The study included 3984 male workers ever employed at the shipyard of Genoa, Italy, between January 1960 and January 1981. The follow-up window ranged from January 1st, 1960 to January 1st, 2015. Data on individual working history were provided by the Personnel Department of the Port Authority of Genoa for all workers included in the study. The data considered for follow-up purposes included: surname and family name, date and place of birth, last known address, date of hire and date of retirement, and job positions held at the shipyard. Vital status was ascertained via record linkage with the Health Registry of the Liguria Region or, for workers who lived out of the Region, through postal follow-up from the Demographic Registry of the last place of residence. For deceased subjects, death certificates were obtained from the Municipality of residence or the Local Health Unit for the period 1960–1987 and from the Mortality Registry of the Liguria Region for the period 1988–2014. Death certificates of workers who lived out of the Liguria Region at the time of death were obtained from the Municipality or the local Health Unit of the last place of residence. The causes of death were classified by an expert coder using the International Classification of Diseases (ICD), 9th Revision. The study was approved by the Regional Ethics Committee (# 042-REG-2016, April 18, 2016). Person years of observation (PY), stratified by five-year age groups and calendar-periods, were computed using STATA statistical software [33]. The expected number of deaths was calculated by applying age, calendar time and cause specific death rates of the male population of the Liguria Region to the PY corresponding to the shipyard cohort. The set of rates was prepared by the Regional Mortality Registry based on mortality and population data provided by the Italian National Institute of Statistics. Standardized Mortality Ratios (SMRs) were computed as the ratio of the observed to the expected number of deaths for overall mortality and specific causes of death. SMRs by time since first hire at the shipyard, length of employment, age at hire and decade of death, and for shipyard trades were also calculated. 95% Confidence Intervals (95%CI) for the SMRs were computed assuming a Poisson distribution for the observed deaths [34]. Locally Weighted Scatterplot Smoother (LOWESS) was used to depict lung and pleural cancer mortality rates across the time points (quinquennia) of the whole follow-up period. LOWESS is a special case of non-parametric regression that allows drawing a smooth curve on a scatter diagram to summarize the relation between variables by making few assumptions about the form of the relationship [35]. Specifically, the expected value of the response variables (i.e., lung or pleural cancer death rates), was estimated, for each calendar period, by applying the weighted least squares method, giving more weight to periods near the calendar period (i.e., quinquennium) whose response was estimated and less weight to periods further away. The number of lung cancer deaths attributable to asbestos exposure (i.e., attributable fraction, AF) [36] was estimated using trade-specific SMRs for pleural cancer as a surrogate of the level of exposure to asbestos. To this aim shipyard trades were combined into five groups according to the cut-off points of the centiles distribution of trade-specific SMRs for pleural cancer. The ratios between lung cancer SMRs for the four higher categories relative to the lowest category (i.e., reference) of pleural cancer were computed to estimate the fraction of lung cancer attributable to asbestos exposure.

## Results

The 3984 workers included in the study contributed to 99,169 PY of observation. The distribution of subjects and PY and their vital status, ascertained as of January 1, 2015, are reported in Table 1 for the whole cohorts and for specific job trades. 638 workers (16%) were alive, 3331 (83.6%) died during the follow-up, and 15 (0.4%) were lost to follow-up. In the whole cohort no increased mortality from all causes was observed (SMR = 99, 95%CI = 96–103) (Table 2) while excesses were detected for all cancers (SMR = 127, 120–134), cancers of the larynx (SMR = 183, 134–244), lung (SMR = 154, 139–170), pleura (SMR = 575, 469–697) and for undefined cancers (SMR = 183, 132–247). The median latency time between date of first hire at the shipyard and date of death for lung cancer and for pleural cancers was 38.7 and 42.8 years, with minimum latency of 9.3 and 6 years, respectively. Four peritoneal neoplasms were observed (SMR = 181, 49–463, data not shown). Increased mortality was detected also for diseases of the respiratory tract (SMR = 127, 114–141), with 29 out of 359 (8.1%) deaths due to asbestosis (SMR = 2277, 1525-3270). Excess mortality was observed for gastrointestinal tract diseases (SMR = 115, 101–131), with 123 out of 215 (57%) deaths from liver cirrhosis (SMR = 136, 113–162), and undefined diseases (SMR = 293, 252–339). Lower than expected mortality was detected for diabetes (SMR = 63, 46–84), diseases of the nervous system (SMR = 70, 52–92), cardiovascular diseases (SMR = 74, 70–79), and for accidental deaths (SMR = 60, 47–75).

**Table 1:** Vital status of shipyard workers employed at the shipyard of Genoa, Italy, between 1960 and 1981 at the end of the follow-up period (1960–2014)

			Alive		Deceased		Lost to follow-up	
Job-titles	No.	PY^a^	No.	(%)	No.	(%)	No.	(%)
Total	3984	99,169	638	(16.0)	3331	(83.6)	15	(0.4)
Fitters	354	9967	82	(23.2)	271	(76.5)	1	(0.3)
Smiths and Shipwrights	229	5378	33	(14.4)	195	(85.2)	1	(0.4)
Plumbers and Coppersmith	365	9586	79	(21.6)	286	(78.4)	–	
Autogenous welders	228	5868	36	(15.8)	191	(83.8)	1	(0.4)
Electrical welders	267	7794	70	(26.2)	197	(73.8)	–	
Electricians	163	4382	19	(11.7)	144	(88.3)	–	
Insulation workers	82	1914	7	(8.5)	75	(91.5)	–	
Ironsmiths	481	9945	67	(13.9)	411	(85.4)	3	(0.6)
Ship demolishers	488	15,158	120	(24.7)	367	(75.2)	1	(0.2)
Stakers	305	7434	40	(13.1)	264	(86.6)	1	(0.3)
Painters	126	2152	1	(0.8)	124	(98.4)	1	(0.8)
Masons	58	1226	5	(8.6)	53	(91.4)	–	
Linoleum, Polishers, Decorators	48	987	4	(8.3)	44	(91.7)	–	
Joiners	172	4016	11	(6.4)	160	(93.0)	1	(0.6)
Carpenters	121	2162	6	(5.0)	114	(94.2)	1	(0.8)
Caulkers	62	954	–		62	(100.0)	–	
Metallurgical workers	242	4088	1	(0.4)	240	(99.2)	1	(0.4)
Careeners	193	6158	57	(29.5)	133	(68.9)	3	(1.6)

^a^) PY: person-years of observation

**Table 2:** Standardized Mortality Ratio (SMR) and 95% Confidence Intervals (95%CI) for all causes and site specific causes of death computed in Genoa shipyard workers (follow-up period 1960–2014)

Cause of death (ICD IX)	Observed	Expected	SMR	95%CI
All causes (0–999)	3331	3360.6	99	96–103
All cancers (140–209)	1184	933.4	127	120–134
Oro-pharynx (140–149)	21	21.6	97	60–148
Esophagus (150)	19	19.5	97	58–151
Stomach (151)	85	81.4	104	83–129
Colon (153)	55	68.1	81	61–105
Rectum (154)	42	33.6	125	90–169
Liver (155)	47	39.2	120	90–159
Pancreas (157)	31	32.9	94	64–133
Larynx (161)	46	25.1	183	134–244
Lung (162)	399	259.5	154	139–170
Pleura (163)	103	17.9	575	469–697
Prostate (185)	72	82.3	88	69–111
Bladder (188)	61	48.6	126	96–162
Undefined (199)	43	23.5	183	132–247
Lymphomas (200–202)	18	21.5	84	50–133
Leukemias (204–208)	20	28.1	71	43–110
Diabetes mellitus (250)	45	71.0	63	46–84
Nervous system diseases (320–389)	52	74.6	70	52–92
Cardiovascular diseases (390–459)	1054	1416.2	74	70–79
Respiratory diseases (460–519)	359	282.6	127	114–141
Asbestosis (501)	29	1.3	2277	1525-3270
Gastrointestinal tract diseases (520–579)	215	186.8	115	101–131
Cirrhosis of the liver (571)	123	90.2	136	113–162
Urogenital system diseases (580–629)	41	63.5	65	47–88
Undefined diseases (780–799)	184	62.8	293	252–339
Accidents (800–999)	74	123.5	60	47–75

Results of the analyses by time since first hire, age at hire, period of hire, and length of employment at the shipyard are shown in Table 3. Excess mortality from all cancers, lung and pleural cancers was inversely related to period of hire (t_trend_ < 0.05). Higher SMRs were observed in workers hired ≤1940 and between 1941 and 1960. Mortality from pleural and laryngeal cancers was inversely related to age at hire (t_trend_ < 0.05); workers first employed at the shipyard at young ages (i.e., ≤34 years old) experienced higher SMRs.

**Table 3:** Standardized Mortality Ratio (SMR) for all causes and site specific causes of death computed in shipyard workers by time since first hire, age at hire, calendar period of hire, and length of employment at the Genoa shipyard

		All Causes		All Cancers		Larynx Cancer		Lung Cancer		Pleural Cancer		Respiratory Diseases	
Covariates	PY	Obs.^a^	SMR	Obs.	SMR	Obs.	SMR	Obs.	SMR	Obs.	SMR	Obs.	SMR
Time since first hire (years)													
0–19	31,802	321	119*	126	136*	5	143	46	152*	11	651*	26	175*
20–29	24,591	492	93	198	122*	7	141	69	141*	11	328*	46	112
30–39	19,222	710	102	270	132*	11	204*	103	176*	21	518*	76	134*
> =40	23,554	1808	97	590	125*	23	205*	181	149*	60	681*	211	124*
*P value t*_*trend*_			*0.051*		*0.521*		*0.324*		*0.941*		*0.274*		*0.319*
Age at hire (years)													
0–24	36,495	1143	97	428	130*	21	242*	148	162*	43	683*	124	124*
25–34	28,273	861	109*	310	147*	13	224*	97	172*	31	788*	80	122
> =35	34,401	1327	96	446	113*	12	113	154	138*	29	378*	155	132*
*P value t*_*trend*_			*0.886*		*0.251*		*0.037*		*0.154*		*0.023*		*0.599*
Period of hire													
< =1940	23,838	1529	119*	444	150*	22	245*	156	215*	37	760*	186	158*
1941–1960	32,009	922	89*	379	124*	9	113	126	143*	39	641*	91	106
1961–1970	11,959	243	73*	110	105	4	159	36	117	8	378*	22	84
> =1971	31,363	637	91*	251	110	11	196	81	119	19	393*	60	115
*P value t*_*trend*_			*0.074*		*< 0.001*		*0.601*		*< 0.001*		*0.009*		*0.071*
Length of employment (years)													
0–9	19,902	416	98	129	104	5	152	46	129	6	234	44	131
10–19	31,572	946	97	348	123*	10	132	108	133*	27	494*	113	141*
20–29	22,921	720	108*	263	145*	10	196	90	180*	29	843*	63	114
> =30	24,774	1249	96	444	129*	21	230*	155	167*	41	638*	139	123*
*P value t*_*trend*_			*0.965*		*0.078*		*0.163*		*0.031*		*0.063*		*0.356*

^a^) Obs.: observed deaths; * *P* < 0.05

Mortality from lung and pleural cancer was directly related to length of employment at the shipyard (t_trend_ 0.031 and 0.063, respectively). Larger excesses were detected in workers employed for 20–29 and ≥ 30 years (lung cancer SMR =180 and 167; pleural cancer SMR =843 and 638).

**Fig. 1:**
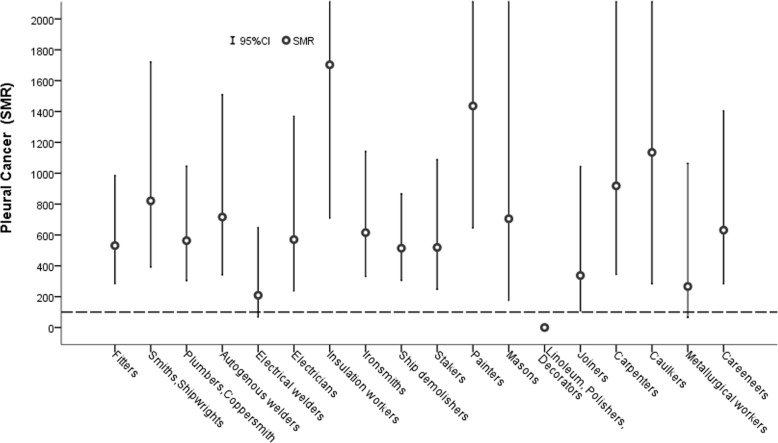
Trade-specific pleural cancer SMR point estimates (○) and 95%CI (vertical bars). Dashed horizontal line indicates SMR = 100; 95%CI are truncated at 2200

**Fig. 2:**
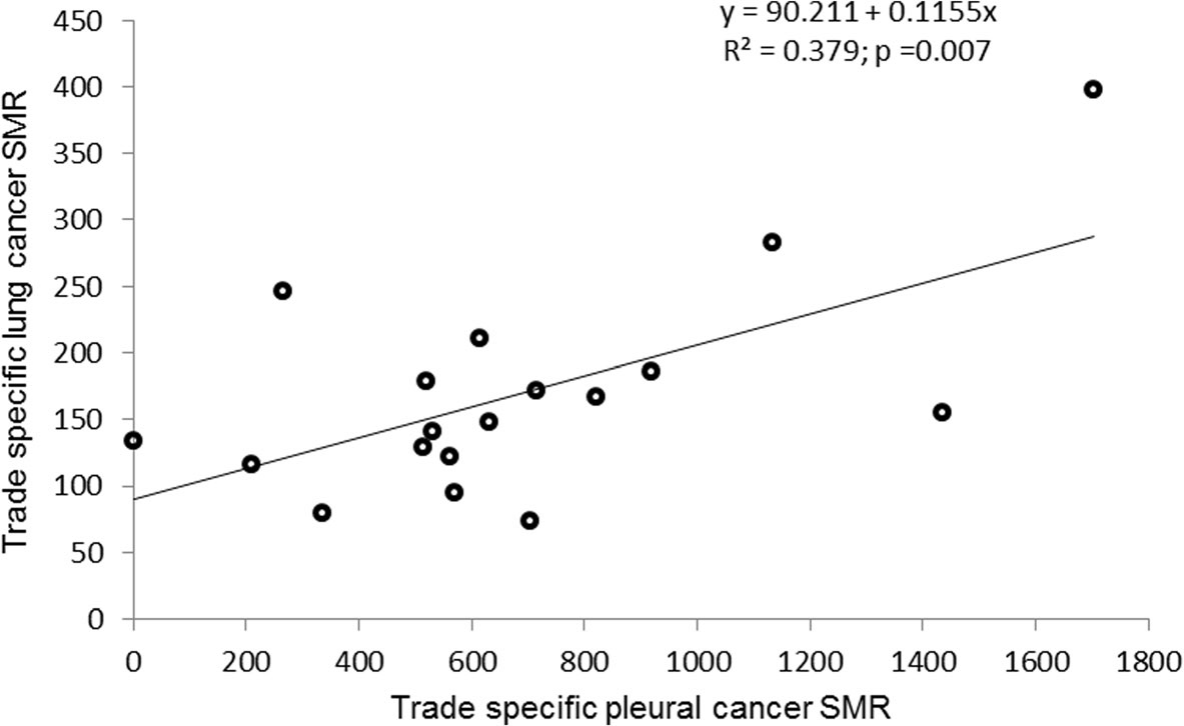
Relationship between trade-specific SMR for pleural and lung cancer

Analysis by job titles (Table 4, Fig. 1), revealed statistically significant increased mortality for all causes in smiths and shipwrights (SMR = 121, 104–138), insulation workers (SMR = 154, 122–192), ironsmiths (SMR = 122, 111–134), painters (SMR = 128, 107–152), and metallurgical workers (SMR = 125, 110–142). Mortality for all cancers was increased among smiths and shipwrights (SMR = 156, 123–196), insulation workers (SMR = 248, 177–340), autogenous welders (SMR= 141, 110-178), ironsmiths (SMR = 159, 134–187), stakers (SMR = 136, 111–165), caulkers (SMR = 172, 108–262), and metallurgical workers (SMR = 172, 137–213). Lung cancer was significantly increased in smiths and shipwrights (SMR = 167, 106–251), insulation workers (SMR = 397, 239–623), ironsmiths (SMR = 211, 157–275), stakers (SMR = 179, 227–246), caulkers (SMR = 283, 131–536), metallurgical workers (SMR = 246, 170–340), and autogenous welders (SMR = 171, 112–251). Excess mortality for laryngeal cancers was detected in insulation workers (722, 184–1965), ironsmiths (390, 198–6952), and stakers (320, 130–6656). Mortality from pleural cancer was increased among all trades but linoleum, polisher and decorator workers (expected deaths in this group was 0.2). The majority of the trade-specific SMRs was > 500 with higher ratios detected among insulation workers (SMR = 1703, 552–3974), painters (SMR = 1436, 524–3126), caulkers (SMR = 1135, 137–4100), carpenters (SMR = 918, 249–2350), and smiths and shipwrights (SMR = 821, 330–1690). Excess mortality for respiratory tract diseases was observed among smiths and shipwrights (SMR = 163, 105–243), insulation workers (SMR = 367, 209–602), ironsmiths (SMR = 170, 127–230), and stakers (SMR = 154, 107–215). A positive relationship was observed between trade-specific SMR for lung cancers and pleural cancers: 38% of the variation of lung cancer SMRs was explained by the variation observed for pleural cancer SMR (R^2^ = 0.379, *p* < 0.05, Fig. 2).

**Table 4:** Trade specific Standardized Mortality Ratio (SMR) for all causes and site specific causes of death among Genoa shipyard workers

	All causes		All cancers		Larynx cancer		Lung cancer		Pleural cancer	Respiratory diseases		
Trades	Obs.^a^	SMR	Obs.	SMR	Obs.	SMR	Obs.	SMR	Obs.	SMR	Obs.	SMR
Fitters	271	83*	104	110	4	166	38	141	10	531*	24	89
Smiths and Shipwrights	195	121*	70	156*	1	79	21	167*	7	821*	22	163*
Plumbers and Coppersmith	286	87*	103	112	2	83	31	122	10	563*	23	83
Autogenous welders	191	114	69	141*	2	151	24	171*	7	716*	16	118
Electrical welders	197	83*	77	108	5	275	24	116	3	209	23	119
Electricians	144	92	50	112	1	85	12	95	5	570*	19	146
Insulation workers	75	154*	36	248*	3	722*	17	397*	5	1703*	14	367*
Ironsmiths	411	122*	142	159*	10	390*	51	211*	10	615*	49	170*
Ship demolishers	367	83*	143	107	4	122	50	129	14	514*	39	111
Stakers	264	106	95	136*	6	320*	35	179*	7	519*	32	154*
Painters	124	128*	32	132	0	0	10	155	6	1436*	15	172
Masons	53	92	15	99	0	0	3	73	2	705	2	39
Linoleum, Polishers, Decorators	44	101	14	126	0	0	4	137	0	0	7	182
Joiners	160	91	47	101	1	80	10	79	3	337	15	98
Carpenters	114	93	35	128	0	0	12	186	4	918*	16	143
Caulkers	62	121	20	172*	2	527	8	283*	2	1135*	6	126
Metallurgical workers	240	125*	79	172*	2	137	29	246*	2	266	25	144
Careeners	133	80*	53	110	3	247	20	148	6	631*	12	89

^a^) Obs.: observed deaths; * *P* < 0.05

The estimated number of lung cancer deaths attributable to asbestos exposure is shown in Table 5. SMRs for lung cancer were significantly increased in workers with SMR for pleural cancer > 265, i.e., the trades used as a reference group in the computation of the lung cancer attributable fraction. The highest RR was detected for trades with the highest SMR for pleural cancer (RR = 1.97, 1.21–3.27) with an estimated AF for lung cancer of 49.3%. Overall, some 90 (22.6%) out of 399 deaths from lung cancer observed in the four groups with SMR for pleural cancer > 265 were attributed to asbestos exposure.

**Table 5:** Lung cancer deaths attributable to asbestos exposure

	Lung cancer				
Pleural Cancer SMR ^a^	Observed	SMR	RR^b^ (95% CI)	Attributable Fraction within exposure stratum	Attributable Deaths^c^ (% of total cases)
< 265	28	119	*reference*	–	–
266–519	124	150	1.26 (0.83–1.98)	20.7%	25.7 (6.4%)
520–615	132	148	1.24 (0.82–1.94)	19.6%	25.9 (6.5%)
616–821	68	154	1.29 (0.82–2.09)	22.7%	15.4 (3.9%)
822–1703	47	235	1.97 (1.21–3.27)	49.3%	23.2 (5.8%)
Total	399	154	–	–	90.2 (22.6%)

^a^)trade-specific SMR for pleural cancer used as a surrogate of the level of exposure to asbestos

^b^)RR = ratio between lung cancer SMR relative to the lowest quintile

^c^)number of lung cancer deaths attributable to each level of pleural cancer SMR

**Fig. 3:**
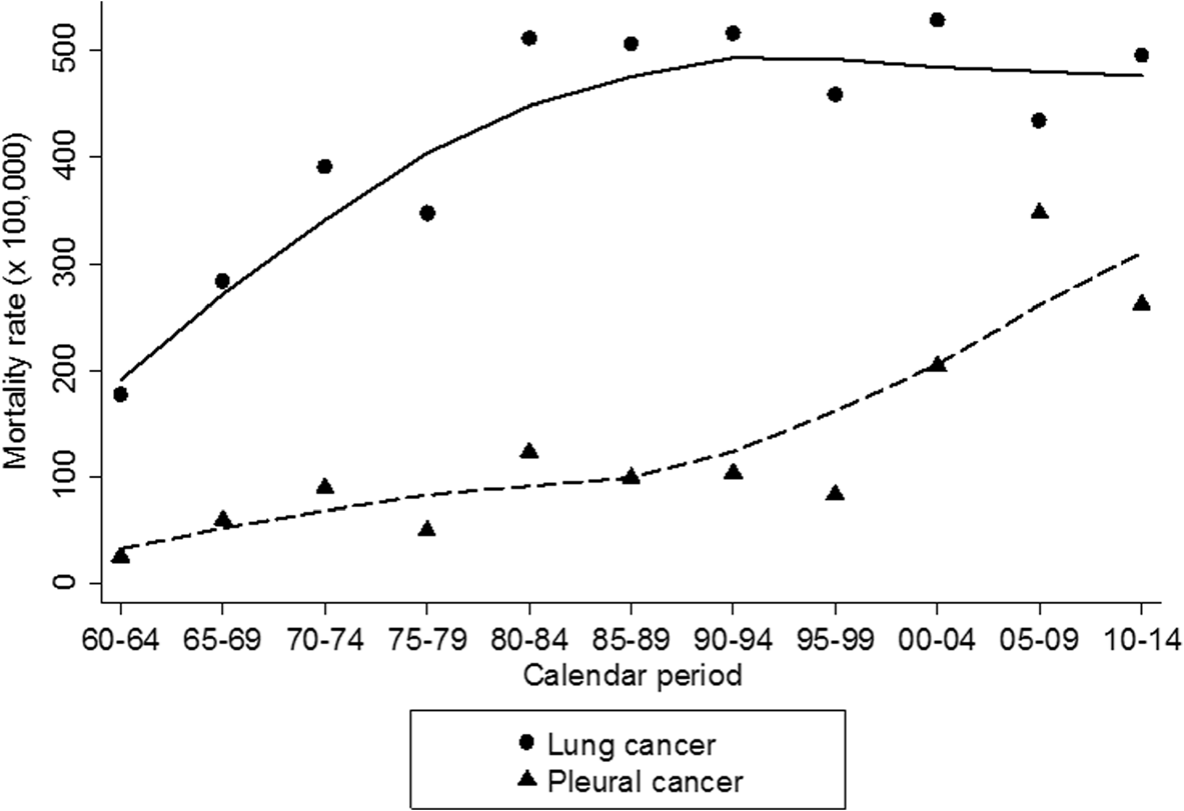
Age adjusted mortality rates by calendar period for lung and pleural cancers. ● Lung Cancer; ▲ Pleural Cancer, a) Lines depict the Lowess estimated trend for death rates across the calendar period (five year intervals)

Lung and pleural cancers mortality rates increased across the follow-up window (Fig. 3). Lung cancer rates increased sharply during the first 30 years of follow-up (1960–1989), reached a plateau during the calendar period (1990–1994) and remained stable until the end of follow-up. Conversely, pleural cancer mortality rates continued to rise across the entire follow-up and more steeply during the last 25 years (i.e., 1990–2014).

## Discussion

The exposure to asbestos at the Genoa shipyard caused significant increased mortality from pleural (+ 475%), lung (+ 54%), and laryngeal cancers (+ 83%), respiratory diseases (+ 27%), asbestosis (+ 2177%), and gastrointestinal tract diseases (+ 15%). These findings confirm earlier results reported for this cohort [30] and are in agreement with data from the literature on the increased risks among shipyard workers [29]. The large increase in mortality from asbestosis and pleural neoplasms is a clear indication of the degree of asbestos exposure in the shipyard before asbestos was definitively banned in Italy in 1994 [19]. Some shipyard trades (e.g., smiths and shipwrights, insulation workers, ironsmiths and painters), showed significantly increased overall mortality, which was explained by the high risks for lung and pleural neoplasms. The risk of pleural neoplasms increased with length of employment and with time since hire at the shipyard. However, the test for trend failed to reach statistical significance. Length of employment was used as a surrogate index of the degree of exposure to asbestos and may not have fully captured exposure intensity given that asbestos levels may have varied across the study period and among shipyard trades. Moreover, the collinearity between length of employment and time since hire (Spearman’s *r* = 0.65) may have masked the true relation with pleural cancers. The observed decreases in mortality for lung and pleural cancer with period of hire is suggestive of high asbestos exposure levels during the early years of the study time frame. This is supported by the modest correlation between age at hire and period of hire (Spearman’s *r* = 0.38). Our findings suggest that the risk for pleural cancer still increases after a latency period of ≥40 years. However, the size of the cohort prevented us from contributing to the scientific debate regarding whether the risk continues to increase [37] or is reduced following a long latency period [38, 39].

Lung cancer rate increased sharply during the first 30 years of follow-up and did not increase any further after 1990. Pleural cancer rate increased slowly during the first 40 years of follow-up (1960–1999) and sharply during the last 25 years (1990–2014). The median latency in our cohort was 42.8 years (minimum latency: 9.3 years), ten years longer than the estimates found in the scientific literature available [40], a difference that is explained by the longer follow-up window of our cohort compared to the 21 studies reviewed by Lanphear and Buncher [41].

Certainly, the association with asbestos does not rule out the effect of other carcinogens. Indeed, it could well be argued that the excess mortality from lung cancer in this cohort could be attributed to higher rate of heavy smoking among shipyard workers than in the reference population and the known synergistic effect with asbestos in causing lung cancer [24] . Yet smoking alone does not explain the excess in lung cancer mortality . In fact, the lack of excess mortality for oropharyngeal (SMR = 97) and esophageal cancers (SMR = 97), together with the 26% deficit in mortality from cardiovascular diseases exclude smoking habits as the only cause of lung cancer, supporting the role of asbestos as a causative agents. As to other occupational carcinogens, the observed excess of lung cancer among autogenous welders (SMR = 171) may be explained by exposure to welding fumes containing polycyclic aromatic hydrocarbons because of work in confined spaces -such as oil tankers- and is unlikely to be explained by a higher proportion of smokers among welders than in the reference population [29, 42–44]. In our study the observed excess mortality for lung cancer correlated with that observed for pleural cancers: 40% of the variation of the SMR for lung cancers observed in shipyard trades was explained by the variability of the SMR for pleural cancers. The variability of pleural cancer risk observed among shipyard trades is likely to reflect differences in the exposure to asbestos. Mean air concentrations of asbestos in shipyards have been reported to range between 0.13 fibers/cm^3^ for pipefitters and 344 fibers/cm^3^ for insulator workers with air level of 896 fibers/cm^3^ measured during removal of pipe lagging [29].

We conclude that our results support the role of asbestos inhalation on lung cancer risk, with 90 (22.6%) out of 399 deaths from lung cancer attributable to inhaled asbestos. The estimated AF was up to 49% for trades heavily exposed to asbestos. However, this estimate is likely to be underrated. The group of trades that were used as a reference had an increased mortality for pleural cancer (SMR up to 265) compared to the death rate of the male population of the Liguria Region, a Region with the highest mortality (i.e., 23.3  × 100.000 males ≥40 years old) among the 38 Italian cancer registries [45]. The estimated AF was based on an internal comparison that is unlikely to be affected by the possible role of other known risk factors for lung cancer, first of all, cigarette smoking. However, the lack of individual data on smoking habits prevented us from directly accounting for the role of smoking. Specifically, in our calculation of the AF we assumed a similar proportion of smokers among shipyard trades, an assumption that could not be proven to hold. We based our calculation of the lung cancer AF on the centiles distribution of trade-specific risks for pleural cancer as those for industrial hygiene were not available. This is a merely statistical approach and is likely to have introduced some degree of uncertainty in the validity of the estimated number of lung cancers attributed to asbestos exposure.

Mortality for laryngeal cancer was also significantly increased (SMR = 183, 134–244). This finding is in agreement with the evidence of a causal role of asbestos reported in the scientific literature [27, 28]. The observed excess risk for cirrhosis of the liver (SMR = 136) suggests a possible role of chronic alcohol abuse -and the interaction with smoking- in the increased mortality from laryngeal cancer. However, the lack of excesses of other alcohol-related cancers (e.g., head and neck, esophageal and oro-pharyngeal cancers), supports a causal relation between asbestos exposure and laryngeal cancer in this cohort.

Projections for the incidence of mesothelioma among males foresaw a peak around 2020 in Europe, and specifically between 2015 and 2024 in Italy [46]. Our study confirms these projections: pleural cancers death rate increased constantly across the follow-up window 1960–2014 with a steep increase during the last 25 years of follow-up when observed rates peaked to values higher than 250 × 100,000.

This historical mortality study has both strengths and limitations. Strengths are the very limited number of workers lost to follow-up (i.e., 0.4%), the long follow-up period that allowed the observation of workers deceased (83.6%.of total workers observed). Possible limitations are the use of “conventional epidemiological indicators” [47] such as length of employment, age at and period of hire, and time since first hire at the shipyard, the lack of industrial hygiene data and measures of environmental levels of asbestos fibers or other occupational agents to characterize workers’ exposure and the lack of individual data on smoking habits. However, these limitations did not prevent the detection of strong associations that are well beyond those possibly due to chance alone or hidden biases.

## Policy implications

Currently, a number of issues still remain unsolved, mainly linked to continual exposure to asbestos fibers in the environment and the likelihood of experiencing such exposure in specific settings (occupational, environmental or both) - more importantly, the related health burden associated to current levels of exposure. Because there is no evidence of a safe threshold of exposure to asbestos and the risk of mesothelioma and lung cancer [21, 48], primary prevention remains the only option to protect workers and the general population - despite those vested interests acting in the name of “Good Science” yet aiming to influence public health policies [49]. The false concept of a safe use of asbestos should be dismissed “tout court” and the use of asbestos be banned worldwide to prevent delayed adverse health effects. Unfortunately, in spite of the accumulated indisputable evidence of asbestos hazards, exposure to asbestos across the globe “remains an international problem” [50–53]. The Indian National Institute of Occupational Health reported early signs of asbestos damage in lungs of current workers employed in the ship-demolishing industry [50]. The use of asbestos poses serious health threat to Asian countries where an epidemic of asbestos related diseases is expected for the next decades [54, 55]. This shows that the massive use of asbestos during the previous century and its environmental persistence, still causes serious health problems and will represent a hazard to humans both in the working and living environments, such as in the cases of asbestos production, import, and use in housing in industrializing countries such as Brazil, India and China [51, 56]. The Hong Kong International Convention for the Safe and Environmentally Sound Recycling of Ships [57], addresses concerns about occupational and environmental issues, including certification and reporting requirement, across many of the world’s ship recycling facilities. However, as of July 2017, such convention has not entered into force yet and has been ratified only by 6 States/Parties worldwide.

The EU Regulation No 1257/2013 on ship recycling [58] represents an attempt to regulate ship recycling/scraping, ensure the proper management of hazardous materials on ships, in order to “prevent, reduce, minimise and, to the extent practicable, eliminate accidents, injuries and other adverse effects on human health and the environment caused by ship recycling”. Such regulation amended Regulation No. 1013/2006 and EC Directive 2009/16 addressing the issue of waste materials which are subject to a transboundary movement for recycling to facilities in Countries that are not members of the Organisation for Economic Cooperation and Development. It also aims to “facilitate the ratification of the 2009 Hong Kong International Convention”. Despite the many regulatory efforts the recent calls for a total ban of asbestos and recommendations for the implementation of preventive and protective efforts to reduce exposure to existing asbestos materials by the International Commission on Occupational Health [59] and the Joint Policy Committee of the Societies of Epidemiology [60] still too little has been done to avoid a next epidemic of asbestos related diseases in industrializing countries. World mine production remains relevant with an estimated world total of 2 million metric tons, half of which produced in Russia and one fifth in China [61]. The World Health Organization has recently estimated that some 125 million people are exposed to asbestos in occupational settings in countries where asbestos use remains unregulated [62] and that more than 107,000 workers exposed to asbestos die every year from lung cancer, mesothelioma and asbestosis [63]. The number of exposed subjects in settings other than the occupational ones remains unknown.

Modern medicine, with its powerful screening and diagnostic tools and the improved therapeutic approaches, including tailored therapy, may contribute to achieve a better cure for asbestos-related lung cancers and, hopefully, mesotheliomas, for which prognosis remains poor and very limited treatment options are available [64–67]. However, a strict public health policy and a strong global political commitment must be adopted and implemented worldwide to prevent asbestos exposure, particularly in developing countries, and to avoid any accidental environmental exposure, including domestic ones that may occur during renovation and demolition of asbestos-containing buildings [68, 69].

## Conclusions

The long follow-up period of our study allowed the detection of a substantial disease burden following exposure to asbestos at the shipyard, confirming the link between asbestos exposure and pleural, lung and laryngeal cancer risks and respiratory diseases including asbestosis. These findings further support the urgent need for the prevention of asbestos related diseases through the effective implementation of asbestos ban and public health policies worldwide to be achieved by a global political commitment, including those countries where asbestos is still mined, manufactured and used.

## Abbreviations

95%CI: 95% Confidence Intervals
AF: Attributable Fraction
ICD: International Classification of Diseases
PY: Person Years
RR: Relative Risk
SMR: Standardized Mortality Ratios
